# Effects of *Saccharomyces cerevisiae* fermentation products on performance and rumen fermentation and microbiota in dairy cows fed a diet containing low quality forage

**DOI:** 10.1186/s40104-017-0167-3

**Published:** 2017-04-28

**Authors:** Wen Zhu, Zihai Wei, Ningning Xu, Fan Yang, Ilkyu Yoon, Yihua Chung, Jianxin Liu, Jiakun Wang

**Affiliations:** 1 Institute of Dairy Science, College of Animal Sciences, Zhejiang University, 866 Yuhangtang Road, Hangzhou, 310058 People’s Republic of China; 2 Diamond V, Cedar Rapids, IA 52405 USA

**Keywords:** Corn stover, Lactating cow, Rumen fermentation, Rumen microbiota, *Saccharomyces cerevisiae* fermentation product

## Abstract

**Background:**

A possible option to meet the increased demand of forage for dairy industry is to use the agricultural by-products, such as corn stover. However, nutritional value of crop residues is low and we have been seeking technologies to improve the value. A feeding trial was performed to evaluate the effects of four levels of *Saccharomyces cerevisiae* fermentation product (SCFP; Original XP; Diamond V) on lactation performance and rumen fermentation in mid-lactation Holstein dairy cows fed a diet containing low-quality forage. Eighty dairy cows were randomly assigned into one of four treatments: basal diet supplemented with 0, 60, 120, or 180 g/d of SCFP per head mixed with 180, 120, 60, or 0 g of corn meal, respectively. The experiment lasted for 10 wks, with the first 2 weeks for adaptation.

**Results:**

Dry matter intake was found to be similar (*P* > 0.05) among the treatments. There was an increasing trend in milk production (linear, *P* ≤ 0.10) with the increasing level of SCFP supplementation, with no effects on contents of milk components (*P* > 0.05). Supplementation of SCFP linearly increased (*P* < 0.05) the N conversion, without affecting rumen pH and ammonia-N (*P* > 0.05). Increasing level of SCFP linearly increased (*P* < 0.05) concentrations of ruminal total volatile fatty acids, acetate, propionate, and butyrate, with no difference in molar proportion of individual acids (*P* > 0.05). The population of fungi and certain cellulolytic bacteria (*Ruminococcus albus*, *R. flavefaciens* and *Fibrobacter succinogenes*) increased linearly (*P* < 0.05) but those of lactate-utilizing (*Selenomonas ruminantium* and *Megasphaera elsdenii*) and lactate-producing bacteria (*Streptococcus bovis*) decreased linearly (*P* ≤ 0.01) with increasing level of SCFP. The urinary purine derivatives increased linearly (*P* < 0.05) in response to SCFP supplementation, indicating that SCFP supplementation may benefit for microbial protein synthesis in the rumen.

**Conclusions:**

The SCFP supplementation was effective in maintaining milk persistency of mid-lactation cows receiving diets containing low-quality forage. The beneficial effect of SCFP could be attributed to improved rumen function; 1) microbial population shift toward greater rumen fermentation efficiency indicated by higher rumen fungi and cellulolytic bacteria and lower lactate producing bacteria, and 2) rumen microbial fermentation toward greater supply of energy and protein indicated by greater ruminal VFA concentration and increased N conversion. Effects of SCFP were dose-depended and greater effects being observed with higher levels of supplementation and the effect was more noticeable during the high THI environment.

## Background

The increase in human consumption of milk has increased the demand for high quality forages for lactating dairy cows. However, China does not have enough high quality forages for dairy cows. A possible option to meet the increased demand of forage is to use the agricultural by-products that are generated in large amounts globally, with estimated 265 million tons of corn stover produced annually in China [1]. These by-products are rich in carbohydrates representing a large potential dietary energy source for ruminants. However, nutritional value of crop residues is low due to their high content of fiber, low digestibility, and low contents of crude protein (CP), metabolizable energy (ME), minerals, and vitamins [2]. Therefore, it is essential to develop nutritional strategies to maintain high milk production when crop residues are used as the main forage source. Feeding ruminal fermentation modifiers has been shown as a cost-effective and safe way to maximize feed utilization of low-quality forage, and thereby improve milk production [3].

*Saccharomyces cerevisiae* fermentation product (SCFP; Original XP; Diamond V, Cedar Rapids, IA, USA) is one of the most widely used rumen fermentation modifiers. A recent meta-analysis of dairy cow studies indicated that SCFP increased milk yield and feed utilization of lactating dairy cows [4]. These effects may be the results of SCFP favorably altered ruminal microbial fermentation, by stimulating the growth and activity of fiber-digesting bacteria, increasing fiber digestion, increasing microbial protein (MCP) synthesis, stimulating growth of lactate-utilizing bacteria; and decreasing accumulation of lactate [5–8]. It is reported that SCFP improved the rumen fermentation of both low quality forages and their mixed diets by stimulating the number of fiber-digesting rumen microbes, especially fungi populations in vitro [9]. It was hypothesized that SCFP may be beneficial when cows are fed low quality forages, by promoting a better forages utilization and therefore by improving nutrient availability. Thus, the objective of the current study was to investigate the effects of several levels of SCFP on dry matter intake (DMI), milk production, rumen fermentation and microbial communities in dairy cows fed a diet containing low quality forage.

## Methods

### Animals

The Animal Care Committee of Zhejiang University approved the use of animals for this experiment (Hangzhou, China). The experiment was conducted as a randomized complete block design with repeated measurements. Eighty Holstein cows (initial mean ± SD: parity 3.23 ± 0.81, 655 ± 65 of kg BW, 180 ± 45 d in milk (DIM), 26.6 ± 0.79 kg/d milk) were divided into 20 blocks based on milk yield and DIM and then randomly assigned within block to one of four treatments. The cows were fed individually and supplemented with 0 (control), 60, 120, or 180 g/d of SCFP per head mixed with 180, 120, 60, or 0 g of corn meal, respectively. The SCFP (Original XP), a fully fermented yeast culture containing residual yeast cells, fermentation metabolites and growth media, was from Diamond V (Cedar Rapids, Iowa, USA). Daily, SCFP was individually top-dressed with 1/3 of the supplement provided at each of the 3 feedings. Each cow was observed for 20 min after feeding to ensure complete consumption of the supplements.

### Diets

Diets were designed according to nutrient requirements for mid-lactation Holstein cows weighing 600 kg and producing 30 kg/d of milk [10]. The ingredients and chemical composition of the experimental diet are presented in Table 1. The forage was comprised (DM basis) of 150 g/kg corn stover (pelletized), 70 g/kg Chinese ryegrass, and 173 g/kg corn silage. These forage sources were considered to be of low quality based on their chemical composition as described in the footnote of Table 1. Samples of forage and concentrate were collected weekly and analyzed to adjust diets to account for DM fluctuation. Feed was offered ad libitum to allow for 10% orts.

**Table 1:** Ingredient and chemical composition of basal diets used in the experiment

Items	Contents
Ingredient, g/kg DM	
Corn silage^a^	173
Chinese ryegrass^a^	70
Corn stover (pelletized)^a^	150
Ground corn	148
Steam-flaked corn	74
Barley	49
Soybean meal	123
Cottonseed meal	49
Beet pulp	85
Brewer’s grains	28
Calcium Carbonate	2
Premix^b^	49
Chemical composition, g/kg DM	
OM	925
CP	149
NDF	415
ADF	246
Non-fibrous carbohydrates (NFC)^c^	346
Ca	6.8
P	4.6
NE_L_^d^, Mcal/kg DM	1.57

^a^Chemical compositions (% of DM) of forages are as follows (*n* = 5): corn silage: OM 94.7, CP 8.1, NDF 69.5, ADF 34.0, and NFC 14.6; Chinese ryegrass: OM 92.3, CP 7.72, NDF 67.5, ADF 42.6, and NFC 16.5; corn stover (pelletized): OM 90.0, CP 6.0, NDF 56.8, ADF 26.7, and NFC 28.8

^b^Formulated to provide (DM basis): 1% CP, 15% ether extracts, 6% crude fiber, 7% Ca, 1.3% P, 10% salt, 3% Mg, 1.5% K, 1% Met, 260 mg/kg Cu, 260 mg/kg Fe, 1,375 mg/kg Zn, 500 mg/kg Mn, 112,000 IU/kg vitamin A, 29,500 IU/kg vitamin D_3_, and 700 IU/kg vitamin E

^c^NFC = 100 - %NDF - %CP - %EE - %ash

^d^Net energy for lactation, calculated based on Ministry of Agriculture of P.R. China recommendations [10]

### Experimental design

The feeding trial was conducted for 8 wk, following a 2-week adaptation when cows were fed a common basal total mixed ration (TMR) (Table 1) without SCFP. Cows were housed in a tie-stall barn and fed 3 times daily at 0630, 1330 and 2000 h with free access to drinking water. Cows were milked 3 times daily at 0700, 1400 and 2030 h. The basal TMR was mixed on site at every feeding, with the grain mix prepared every 2 wk.

### Sampling, measurement, and analyses

In order to calculate the temperature-humidity index (THI), temperature and relatively humidity (RH) inside the barn were measured as described by Zhu et al. [11]. The THI was calculated as: THI = TD – (0.55-0.55 RH/100) (TD-58), where TD was the dry bulb temperature in °F (°F = 32 + 1.8°C) and RH was expressed as a percentage [12]. The average daily THI were calculated.

Feed offered and refused were weighed daily for each cow. Representative samples (1 kg) of all dietary ingredients, TMR and orts were collected on d 14 of the adaptation period and d 3 of each week during the experimental period. Sample preparation and analysis were performed according to Zhu et al. [13]. All samples were analyzed for contents of DM, CP, crude ash and acid detergent fiber (ADF) according to AOAC methods as described by procedures #934.01, #988.05, #927.02, and #973.18, respectively, and neutral detergent fiber (NDF) was assayed without a heat stable amylase and inclusive of residual ash [14, 15].

Milk samples were collected at each milking on d 11 to 14 of the adaptation period and on d 4 and 5 of each week during the experimental period. At each milking 50 mL milk samples were collected and pooled in a proportion of 4:3:3 considering the ratio of milk yield for the 3 times milking. Potassium dichromate (milk preservative, D&F Control Systems, San Ramon, CA, USA) was added at 0.6 g/kg to milk samples prior to storage. Milk samples were sent to Shanghai Dairy Herd Improvement testing center (Shanghai, China) for analysis of milk fat, protein, lactose, somatic cell count, and milk urea nitrogen by infrared analysis using a spectrophotometer (Foss-4000, Foss, Hillerød, Denmark) [16].

Rumen fluid (approximately 100 mL) was sampled using a stomach tube from 40 cows randomly (10 per treatment) at approximately 3 h after the morning feeding on d 7 of wk 4 and 8 during the experimental period as described by Shen et al. [17]. Fifty milliliters of collections were squeezed through 4 layers of cheesecloth, and rumen pH was measured immediately using a portable pH meter (Starter 300, Ohaus Instruments Co. Ltd., Shanghai, China). Two 5.0 mL subsamples of each rumen filtrate were collected and stored at -20°C for further analysis of ammonia nitrogen (N) and volatile fatty acids (VFA). One subsample was acidified with 1.0 mL of 250 g/kg orthophosphoric acid for VFA determination. Concentrations of VFA were determined by gas chromatography (GC-8A, Shimadzu Corp., Kyoto, Japan) according to the method described by Chaney and Marbach [18]. Another subsample was used to determine the ammonia-N by colorimetric method, as described by Hu et al. [19]. Another 50 mL of unfiltered rumen fluid was stored at -80°C for DNA extraction to determine the relative quantity of ruminal fibrolytic bacteria, fungi, protozoa, and lactate-utilizing and lactate-producing bacteria to 16S rDNA of total bacteria (as described below).

Cow body weight (BW) was estimated on two consecutive days at the beginning and end of the experiment based on the measurement of heart girth and body length using the following equation: Estimated BW (kg) = heart girth^2^ (m) × body length (m) × 90 [20]. A scaled score (5-point scale, where 1 = thin and 5 = fat) was used to determine body condition score (BCS) on d 7 of wk 2, 4, 6, and 8 by 2 experienced investigators blinded to treatments according to Edmonson et al. [21]. Values from investigators were averaged for each scoring date and the mean BCS were used for statistical analysis.

### Estimation of microbial protein synthesis in the rumen

Microbial protein (MCP) synthesis in the rumen was estimated by urinary purine derivatives (PD) as reported by Chen and Gomes [22]. Spot urine samples (10 mL each time) were collected twice daily at approximately 3 h and 6 h after the morning feeding on d 6 of wk 4 and 8 during the experimental period. The daily urine samples were pooled at equal portion by cow and 15 mL of subsamples were acidified immediately with 60 mL 0.036 mol/L H_2_SO_4_ (1:4) and stored at -20°C for later analysis. For analysis of PD, allantoin and uric acid were analyzed by the procedure of Chen and Gomes [22]. Creatinine was detected using a picric acid assay [23]. Creatinine was used as the marker to estimate urine volume, and was assumed to be excreted at a rate of 29 mg/kg of BW for calculating the urine volume excretion rate [24, 25].

### Determination of rumen microbial population

A total of 2 mL of unfiltered rumen fluid was used for the genomic DNA extraction with the RBB + C method as described by Yu and Morrison [26]. The RNA and protein were removed by sequential digestion with RNase A and proteinase K. Then DNA was purified using columns from the QIAamp DNA Stool Mini Kit (QIAGEN, Valencia, CA, USA). The purity and concentration of total DNA were determined by spectroscopy (NanoDrop 2000, Thermo Scientific Inc., Wilmington, DE, USA), and the extracted DNA was diluted to 10 ng/μL. The integrity of the total DNA was assessed by 10 g/kg agarose gel electrophoresis.

Quantitative real-time PCR was used to determine the relative abundance of protozoa, fungi and 6 bacterial populations using previously validated primers listed in Table 2 [27–32]. The real-time qPCR assays were performed on an ABI 7500 Real-Time PCR System (Applied Biosystems, Foster City, CA, USA) using a kit (SYBR Premix Ex Taq kit; TaKaRa Biosystems Co. Ltd, Dalian, China). The template DNA used possessed an A_260_/A_280_ ratio within the range of 1.7 to 1.9. The qPCR assays was performed in a total volume of 20 μL solution contained 1 μL of genomic DNA (10 ng/μL), 0.2 μmol/L of each primer, 10 μL of SYBR Premix Ex Taq (2×), 0.4 μL of ROX II (50×), and double-distilled water. All qPCR assays were performed in triplicate for each sample. For all primers, amplification started with a denaturalization at 94°C for 10 s followed by 40 cycles of 95°C for 5 s and 60°C for 34 s.

**Table 2:** Primers used in this study

Target		Primer sequences (5′→3′)	Product size, bp	Reference
Total bacteria	F	CGGCAACGAGCGCAACCC	130	[27]
	R	CCATTGTAGCACGTGTGTAGCC		[27]
Protozoa	F	GCTTTCGWTGGTAGTGTATT	223	[28]
	R	CTTGCCCTCYAATCGTWCT		[28]
Fungi	F	GAGGAAGTAAAAGTCGTAACAAGGTTTC	120	[27]
	R	CAAATTCACAAAGGGTAGGATGATT		[27]
*Ruminococcus albus*	F	CCCTAAAAGCAGTCTTAGTTCG	175	[29]
	R	CCTCCTTGCGGTTAGAACA		[29]
*R. flavefaciens*	F	CGAACGGAGATAATTTGAGTTTACTTAGG	132	[27]
	R	CGGTCTCTGTATGTTATGAGGTATTACC		[27]
*Fibrobacter succinogenes*	F	GTTCGGAATTACTGGGCGTAAA	121	[27]
	R	CGCCTGCCCCTGAACTATC		[27]
*Selenomonas ruminantium*	F	TGCTAATACCGAATGTTG	515	[30]
	R	TCCTGCACTCAAGAAAGA		[30]
*Megasphaera elsdenii*	F	GACCGAAACTGCGATGCTAGA	128	[31]
	R	CGCCTCAGCGTCAGTTGTC		[31]
*Streptococcus bovis*	F	ATGTTAGATGCTTGAAAGGAGCAA	90	[32]
	R	CGCCTTGGTGAGCCGTTA		[32]

### Calculations and statistical analysis

The quantification of protozoa, fungi, *Ruminococcus albus*, *R. flavefaciens, Fibrobacter succinogenes, Selenomonas ruminantium, Megasphaera elsdenii, and Streptococcus bovis* was expressed as a ratio to 16S rDNA of total bacteria. The 2^−ΔΔCT^ method was used to analyze the relative changes in each gene expression, where Ct represented threshold cycle [33].

Data analyses were carried out using SAS software (version 9.0, SAS Institute Inc., Cary, NC, USA) [34]. All data except for BW gain were analyzed through the PROC MIXED program of SAS with the covariance type AR (1) for repeated measures. To determine differences in lactation performance [DMI, milk yield, ECM, and feed efficiency] among treatments, calculated mean values of DMI and milk yield on d 11 to 14 of the 14-d adaptation period were used as the covariate for treatment response. A randomized block design with repeated measurements was used. The model included week, treatment, interaction of treatment × week and block as fixed effects. Cow was included as a random effect to provide the error term to test the significance of the differences. Means were separated using the PDIFF option in the LSMEANS statement.

Data on BW gain were analyzed using the PROC GLM of SAS. The statistical model was the same as indicated above except that week and treatment × week were omitted. Linear and quadratic effects of treatment were tested for all data using orthogonal polynomial contrasts.

Results are reported as least squares means. Probability values of *P* ≤ 0.05 were defined as statistically significant and the values 0.05 < *P* ≤ 0.10 were defined as trend.

## Results

### Feed intake and lactation performance

**Fig. 1:**
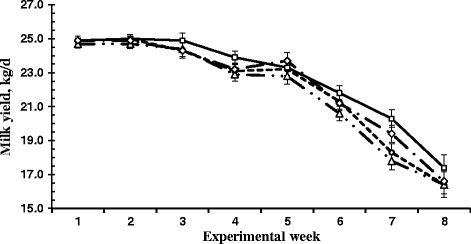
Change in milk yield of lactating cows fed a diet containing low-quality forage with supplementation of a *Saccharomyces cerevisiae* fermentation products at 0 (○), 60 (∆), 120 (□), or 180 (◊) g/d. Bars indicate standard error of mean

**Fig. 2:**
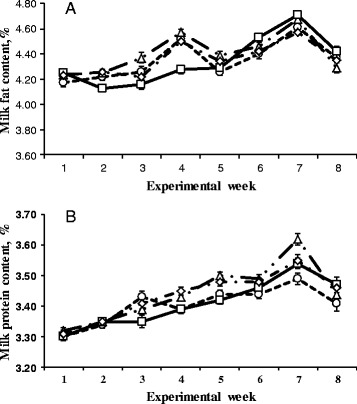
Change in contents of milk fat (**a**) and milk protein (**b**) in lactating cows fed a diet containing low-quality forage with supplementation of a *Saccharomyces cerevisiae* fermentation products at 0 (○), 60 (∆), 120 (□), or 180 (◊) g/d. Bars indicate standard error of mean

Body weight gain and BCS were not affected (*P* > 0.05) by SCFP supplementation. Dry matter intake was similar (*P* > 0.05) among the treatments. Milk yield had an increasing trend (linear, *P* = 0.08) with the increasing amount of SCFP supplementation (Table 3). Energy-corrected milk (ECM) was not affected by SCFP supplementation (*P* > 0.05). Feed efficiency (ECM/DMI) was not affected (*P* > 0.05), whereas N conversion (milk protein yield/dietary CP intake) increased linearly (*P* < 0.05) with the increasing amount of SCFP. Contents of milk components were similar (*P* > 0.05) among the treatments. Treatment × week interactions for milk yield (Fig. 1) and contents of milk fat and milk protein (Fig. 2) with an enhanced SCFP effect being found when cows were supplemented to SCFP for an extended period of time.

**Table 3:** Effects of *Saccharomyces cerevisiae* fermentation product on lactation performance of dairy cows fed corn stover-containing diet

Item^a^	SCFP^b^, g/d				SEM	*P*-value^c^			
	0	60	120	180		L	Q	W	T × W
DMI, kg/d	20.1	20.3	19.9	19.9	0.22	0.26	0.54	<0.01	0.92
Milk yield, kg/d	22.1	21.8	22.6	22.3	0.28	0.08	0.89	<0.01	<0.01
ECM, kg/d	25.4	25.8	26.0	25.7	0.35	0.52	0.35	<0.01	<0.01
Milk composition, %									
Fat	4.35	4.41	4.35	4.36	0.075	0.95	0.81	<0.01	<0.01
Protein	3.41	3.44	3.41	3.44	0.039	0.74	0.87	<0.01	<0.01
Lactose	4.78	4.79	4.73	4.75	0.036	0.33	0.87	<0.01	0.65
Total solids	13.7	13.7	13.4	13.6	0.19	0.55	0.59	<0.01	0.98
SCC, ×10^4^	57.9	35.2	46.9	47.6	11.4	0.63	0.78	0.95	0.75
MUN, mg/dL	13.9	13.8	13.9	13.8	0.37	0.84	0.86	<0.01	0.75
Initial BW, kg	654	643	670	651	13.7	0.78	0.47		
BW gain, kg/d	0.17	0.22	0.15	0.05	0.14	0.62	0.27		
BCS	2.98	2.77	2.99	2.80	0.10	0.47	0.94	0.04	0.20
Feed efficiency	1.28	1.28	1.32	1.28	0.025	0.17	0.50	<0.01	0.67
N conversion	24.7	24.3	25.2	25.1	0.21	0.04	0.82	<0.01	0.17

^a^DMI = dry matter intake; ECM (energy-corrected milk, kg) = 0.3246 × milk yield (kg) + 13.86 × fat yield (kg) + 7.04 × protein yield (kg) [44]; SCC = somatic cell counts; MUN = milk urea nitrogen; Feed efficiency = kg of ECM/kg of DMI; BCS = body condition score; N conversion = kg of milk protein yield/kg of dietary crude protein intake × 100

^b^ *Saccharomyces cerevisiae* fermentation product (Diamond V Original XP, Cedar Rapids, Iowa, USA)

^c^T = treatment effect; W = week effect; T × W = interaction between treatment and week; L = linear effect of treatment; Q = quadratic effect of treatment

### Rumen fermentation parameters

Rumen pH and ammonia-N concentration were similar (*P* > 0.05) among the treatments (Table 4). Greater feeding rate of SCFP increased (linear, *P* < 0.01) concentrations of total VFA, acetate, propionate, and butyrate, with no difference (*P* > 0.05) in molar proportion of individual acids and acetate to propionate ratio (Table 4).

**Table 4:** Effects of *Saccharomyces cerevisiae* fermentation product on rumen fermentation characteristics of dairy cows fed corn stover-containing diet

Item^a^	SCFP^b^, g/d				SEM	*P*-value	
	0	60	120	180		Linear	Quadratic
pH	6.31	6.32	6.41	6.30	0.06	0.82	0.32
Ammonia-N, mg/dL	17.2	17.0	16.9	17.1	0.24	0.52	0.39
Total VFA^a^, mmol/L	93.6	109.3	110.4	114.9	4.56	<0.01	0.23
Acetate, mmol/L	63.3	73.6	73.9	81.1	2.80	<0.01	0.59
Propionate, mmol/L	17.4	20.6	20.7	22.5	0.71	<0.01	0.35
Butyrate, mmol/L	12.8	15.7	15.7	16.5	0.52	<0.01	0.05
Molar proportion, mmol/L/100 mmol/L							
Acetate (Ac)	67.8	67.5	66.9	67.1	0.53	0.30	0.60
Propionate (Pr)	18.6	18.8	18.9	19.0	0.33	0.36	0.84
Butyrate	13.7	13.7	14.2	13.9	0.24	0.32	0.42
Ac:Pr	3.66	3.64	3.55	3.53	0.070	0.22	0.86

^a^Total VFA = Total volatile fatty acids, which is the sum of acetate, propionate and butyrate

^b^ *Saccharomyces cerevisiae* fermentation product (Diamond V Original XP, Cedar Rapids, Iowa, USA)

### Rumen microbial population

The population of fungi increased (quadratic, *P* < 0.05) while protozoa decreased (linear, *P* < 0.01; quadratic, *P* < 0.05) with increasing amount of SCFP (Table 5). In response to SCFP supplementation, cellulolytic bacteria, including *R. flavefaciens* (linear, *P* < 0.01; quadratic, *P* = 0.01) and *F. succinogenes* (linear, *P* < 0.01) increased, but *R. albus* (linear, *P* < 0.05) decreased. *M. elsdenii* (linear, *P* < 0.01; quadratic, *P* < 0.01), *S. ruminantium* (linear, *P* < 0.01; quadratic, *P* < 0.01) and *S. bovis* (linear, *P* < 0.01; quadratic, *P* < 0.01) decreased with increasing amount of SCFP.

**Table 5:** Effects of *Saccharomyces cerevisiae* fermentation product on rumen microbial populations of dairy cows fed corn stover-containing diet

Item^a^	SCFP^b^, g/d				SEM	*P*-value	
	0	60	120	180		Linear	Quadratic
Fungi, 10^-3^	1.79	1.89	2.04	1.88	0.063	0.12	0.05
Protozoa, 10^-1^	1.59	1.24	1.30	1.14	0.044	<0.01	0.03
*Ruminococcus albus*, 10^-3^	5.81	5.37	4.86	4.99	0.199	<0.01	0.16
*Ruminococus flavefaciens*, 10^-2^	1.33	1.44	2.69	2.36	0.75	<0.01	0.01
*Fibrobacter succinogenes*, 10^-2^	1.47	1.73	3.04	3.24	0.078	<0.01	0.74
*Selenomonas ruminantium*, 10^-7^	4.23	3.32	3.06	3.03	0.139	<0.01	0.003
*Megasphaera elsdenii*, 10^-5^	17.6	12.7	7.27	12.0	0.52	<0.01	<0.01
*Streptococcus bovis*, 10^-6^	3.84	3.34	2.07	2.92	0.98	<0.01	<0.01

^a^ Rumen microbial population was expressed as the ratio to total bacterial 16S rDNA

^b^ *Saccharomyces cerevisiae* fermentation product (Diamond V Original XP, Cedar Rapids, Iowa, USA)

### Estimated MCP synthesis in the rumen

Estimated urine volume was not influenced by the SCFP supplementation (*P* > 0.05, Table 6). Concentrations of uric acid and endogenous PD were similar (*P* > 0.05) among the treatments. Allantoin (linear, *P* = 0.01) concentration and the sum of urinary PD (linear, *P* = 0.05) increased linearly with the increasing amount of SCFP. Thus, the ruminal MCP estimated from urinary PD increased (linear, *P* < 0.01) with the increasing amount of SCFP, with 12.8% or 166 g/d higher MCP in the cows fed 120 g/d SCFP than that in the control.

**Table 6:** Effects of *Saccharomyces cerevisiae* fermentation product on rumen microbial protein (MCP) supply of dairy cows fed corn stover-containing diet

Item^a^	SCFP^b^, g/d				SEM	*P*-value	
	0	60	120	180		Linear	Quadratic
Urine volume^a^, L/d	23.0	22.9	22.1	22.3	0.96	0.54	0.90
Urinary PD^b^, mmol/d							
Allantoin	304	280	351	330	12.6	0.01	0.90
Uric acid	27.0	28.0	25.3	27.3	1.15	0.74	0.68
Endogenous PD	50.6	45.2	47.7	44.7	3.97	0.63	0.84
Sum	330	307	373	346	12.7	0.05	0.89
MCP, g/d	1,293	1,120	1,459	1,418	47.6	<0.01	0.59

^a^Urine volume (L/d) = body weight (kg) × 29 (mg/d)/creatinine (mg/L) [25]; Endogenous purine derivatives (PD) = 0.385 × BW^0.75^; Sum = allantoin + uric acid − endogenous PD; Microbial protein (MCP), indirectly calculated based on PD [22]

^b^ *Saccharomyces cerevisiae* fermentation product (Diamond V Original XP, Cedar Rapids, Iowa, USA)

## Discussion

**Fig. 3:**
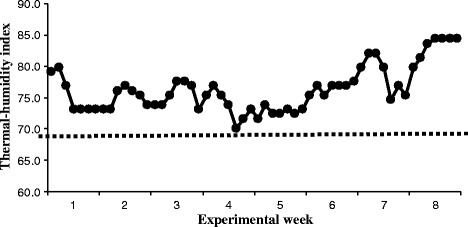
Daily mean thermal-humidity index (THI) during the trial period. Dashed line represents THI = 68, when cows are expected to suffer from heat stress [35]

There was a linear trend of increasing milk yield in response to SCFP supplementation in the present study, in agreement with the results of the meta-analysis by Poppy et al. [4], where an overall positive effect of SCFP on milk yield was reported. One noticeable observation is that the positive effect of SCFP on milk production was more apparent during the second half (wk 5 to 8) of the study (Fig. 1). It may suggest that cows need to adapt to the supplement for several weeks before production response is detectable. Another possible explanation is the changes in weather pattern during the trial period. Weather became hot from the sixth week of the study and the average daily mean thermal-humidity index [35] started moving upward (Fig. 3) while milk production decreased sharply during that time (Fig. 1). The heat wave may cause additional stress to dairy cows particularly when they were consuming diets containing low quality forages, which contributed to the decreased milk production. The lactation curve for the last four weeks showed that milk production in cows fed 120 and 180 g/d SCFP declined slower than that of the control. This suggests that increasing levels of SCFP might be more effective in maintaining milk persistency of mid-lactation cows under hot environment. Improved feed efficiency has been reported consistently when mid-lactation dairy cows were supplemented with SCFP during summer months [11, 36]. In our study, feed efficiency of SCFP supplemented cows was more apparent as the length of the supplementation was extended.

Improving rumen microbial community, especially those that are involved in fiber digestion would be the key components for maintaining productivity of lactating dairy cows when fed low quality forages. The increased total VFA concentrations with SCFP supplementation indicated stimulated rumen microbial fermentation activities, and help support the milk production and persistency as was observed in the present study. Increased VFA production could be attributed to an increased rumen fungi and fiber-digesting bacteria population. In vivo and in vitro studies have documented positive effects of SCFP on rumen fermentation [9, 37]. Increases in rumen propionate have also been observed in previous studies when SCFP was fed [5, 38]. Higher propionic acid concentrations observed with SCFP in the current study would lead to an increased glucogenic potential of the diet and milk production.

It is reported that variable response in VFA production and pattern with yeast culture supplementation is a consequence of yeast culture’s effect on the growth of different species of rumen microbes [39]. Rumen microbial populations detected in the current study were altered in response to SCFP supplementation. Supplementation with SCFP could provide various growth factors, pro-vitamins, and/or micronutrients that help stimulate the growth of ruminal bacteria [40]. Vitamins such as biotin and thiamine that could be provided by SCFP supplementation are reported to be required for fungal growth and activity [41]. In the current study, supplementation of SCFP increased the rumen fungi population, and similar results were reported in a previous in vitro study [9]. In the present study, SCFP supplementation stimulated the growth of cellulolytic bacterial population (*R. flavefaciens* and *F. succinogenes*). Such effects have also been reported in earlier studies [8, 37]. The reduction of lactate-producing bacteria (*S. bovis*) in the present study could contribute to the stability of rumen fermentation. The stabilized rumen condition allows the increased growth and activity of fiber-digesting bacteria, resulting in increased ruminal VFA concentrations [5]. The decrease in number of protozoa in response to SCFP supplementation may decrease the bacterial preying and allows more microbial protein to reach the small intestine. It is reported that steers supplemented with SCFP decreased the proportion of Entodinium from 87.7 to 69.6% [42]. Entodinium is a rumen protozoa responsible for engulfing bacteria and reducing microbial protein supply to the small intestine [43]. Microbial protein is a high quality protein source for dairy cows, and was increased in response to SCFP supplementation in our study. Such effects have been reported in some other studies [7, 9].

Overall, SCFP supplementation manipulated rumen microbial population that resulted in improved energy supply (enhanced VFA production) and improved protein nutrition (greater microbial protein synthesis and more efficient conversion of dietary N to milk N) of lactating cows consuming diets containing low quality forages.

## Conclusion

Supplementation of SCFP shifted rumen microbial population to a greater energetic and nitrogen efficiency of dairy cows consuming diets containing low quality forages. These changes with SCFP supplementation support maintaining better milk persistency of mid-lactation cows particularly when cows were under hot environment. The effects of SCFP were dose-dependent and greater effects being observed with higher levels.

## Abbreviations

ADF: Acid detergent fiber
BW: Body weight
CP: Crude protein
DIM: Days in milk
DM: Dry matter
MCP: Microbial crude protein
ME: Metabolizable energy
N: Nitrogen
NDF: Neutral detergent fiber
PD: Purine derivatives
SCFP: *Saccharomyces cerevisiae* fermentation product
TMR: Total mixed ration
VFA: Volatile fatty acids
